# Factors Contributing to Short‐Term Structural Variability in a Longitudinal MRI Dataset

**DOI:** 10.1002/hbm.70500

**Published:** 2026-03-12

**Authors:** Polona Kalc, Mayla ter Veer, Robert Dahnke, Gabriel Ziegler, Simone Kühn, Christian Gaser

**Affiliations:** ^1^ Structural Brain Mapping Group, Department of Neurology Jena University Hospital Jena Germany; ^2^ Department of Biological Psychology and Cognitive Neuroscience Institute for Psychology, Friedrich‐Schiller University of Jena Jena Germany; ^3^ Department of Psychiatry and Psychotherapy Jena University Hospital Jena Germany; ^4^ German Center for Mental Health (DZPG), Jena‐Halle‐Magdeburg Germany; ^5^ German Center for Neurodegenerative Diseases (DZNE) Magdeburg Germany; ^6^ Institute of Cognitive Neurology and Dementia Research (IKND), Otto‐von‐Guericke University Magdeburg Germany; ^7^ Center for Environmental Neuroscience, Max Planck Institute for Human Development Berlin Germany

**Keywords:** longitudinal, partial least squares, structural MRI, within‐subject variance

## Abstract

When planning longitudinal magnetic resonance imaging (MRI) studies, it is advisable to consider various (confounding) factors that could influence brain structural changes over time. The goal of this study was to identify factors that contribute to intraindividual variability of brain structure within a short period of time. We employed multilevel sparse partial least squares regression to investigate the changes in regional gray matter volume in the longitudinal Day2day MRI dataset. The findings suggest that the changes in regional GM volume estimations were primarily driven by image quality, while the outdoor temperature and time since baseline appeared as the main predictors of volumetric changes in insular and diencephalic brain regions. We additionally investigated factors associated with variability in image quality. The findings underscore the importance of maintaining adequate participant arousal during scanning.

## Graphical abstract

We applied a multilevel sparse PLS model to identify factors associated with short‐term, intraindividual changes in the regional gray matter volume of the human brain, using a longitudinal MRI Day2day dataset. Our analyses showed that aspects of image quality beyond visible motion artifacts had a significant impact on the results.

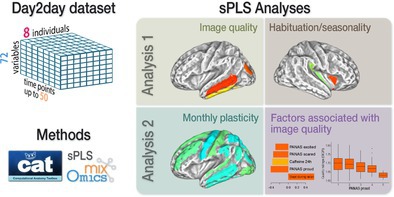

## 1 Introduction

Brain plasticity has been the subject of intensive study over the past decades (Lövdén et al. 2013; May 2011; May et al. 2007), with longitudinal MRI studies proving essential for unraveling patterns of change in brain development and aging (Giedd et al. 1999; Hedman et al. 2012; Raz et al. 2005; Resnick et al. 2003; Strike et al. 2023). One of the assumptions underlying brain imaging studies is that MRI scans provide a reliable measure of brain structure (Vidal‐Piñeiro et al. 2024). Accordingly, two scans of the same individual—along with any derived features—acquired within a short temporal interval should exhibit only minimal differences. However, this is not necessarily the case, due to measurement errors, imaging side effects and actual minor anatomical changes in the brain that can occur within days or even hours (Karch et al. 2019; Kodama et al. 2018; Månsson et al. 2020). Though often overlooked, intraindividual investigations offer insights into short‐term fluctuations and subtle morphometric changes of the brain.

The investigation of short‐term structural changes in the brain has gained increasing attention, with several studies highlighting factors that may contribute to rapid neural adaptations. It has been demonstrated that daily physiological variations that occur under nonexperimental conditions can predict short‐term alteration of brain structure and function, such as water (Duning et al. 2005; Kempton et al. 2009) and caffeine intake (Field et al. 2003; Koppelstaetter et al. 2010), exercise (Erickson et al. 2011; Thomas et al. 2012) or the menstrual cycle (Hagemann et al. 2011; Heller et al. 2025; Lisofsky et al. 2015; Pritschet et al. 2020).

May (2011) emphasized the importance of examining variables outside the experimental conditions that may influence brain plasticity to rule out systematic bias. So far, only a few studies have investigated the role of factors that may predict short‐term variability of brain structure within a person. Karch et al. (2019) found that the number of days since the first scan and the time of the day during scanning are two robust predictors of within‐person variability in brain structure. They additionally identified that testosterone levels and steps taken on the day before the measurement were predictors of intraindividual variability in gray matter (GM) volume in the Day2day dataset (Karch et al. 2019). Building upon the results of studies conducted with the Day2day dataset (Filevich et al. 2017; Karch et al. 2019; Kühn et al. 2022), the present study aimed to identify variables that predict intraindividual changes in brain structure over the course of a few days. In contrast to the work of Karch et al. (2019) who used FreeSurfer and the VBM8 toolbox, the data in this study were processed using the Computational Anatomy Toolbox (CAT12) (Gaser et al. 2024), which showed a more accurate performance in voxel‐based morphometry studies in comparison to the VBM8 toolbox (Farokhian et al. 2017; Matsuda et al. 2012). In this exploratory study, we used highly sensitive multilevel sparse partial least squares regression (Liquet et al. 2012) to identify the most relevant time‐varying variables associated with within‐person structural changes in regional gray matter volume in a high‐frequency longitudinal setting with (on average) more than 30 MRI scans per person over 6 months. This focus on state variability enables the identification of measurement‐ and subject‐specific contributing factors that may influence (undesirable) brain morphometric changes, which should be accounted for in future MRI studies.

## 2 Method

### 2.1 Data

For the purposes of this study, we used the Day2day dataset, which is extensively described in Filevich et al. (2017). Briefly, eight participants (M_Age_ = 29 ± 2.58 years, age range: 24–32 years, two men) took part in the original study, which was conducted from July 2013 to February 2014. Participants were scanned over the course of several months with the aim to acquire two to three scans per week, with interscan intervals differing between and within subjects (Table 1). The average time between two scans was 4.24 days (SD = 4.52 days, min = 1 day, max = 33 days). Across all subjects, 280 measurement points were obtained. The participants were healthy and were not diagnosed with any psychiatric diagnosis.

**TABLE 1:** Characteristics of the sample.

ID	Age	Gender	Days to completion	No. of sessions	Average interscan interval
1	24	Female	168	50	3.4
2	28	Female	107	13	8.2
3	31	Female	394	50	7.9
4	32	Male	56	11	5.1
5	29	Female	208	46	4.5
6	24	Female	170	47	3.6
7	30	Male	218	43	5.1
8	29	Female	232	50	4.6

### 2.2 MRI Data Acquisition

Brain scans were acquired using a 3 Tesla Magnetom Trio MRI scanner system (Siemens Medical Systems, Erlangen, Germany) with a 12‐channel head coil. A three‐dimensional T1‐weighted magnetization prepared gradient‐echo sequence (MPRAGE) with the following parameters was used: TR = 2500 ms, TE = 4.77 ms, TI = 1100 ms, FOV = 256 × 256 × 192, flip angle = 7°, bandwidth = 140 Hz/pixel, 1 mm isotropic voxel size, 9:20 min scan duration.

### 2.3 Image Processing

The T1w images were preprocessed using Computational Anatomy Toolbox (CAT12, version 12.9 r2560), which is an extension to SPM12 (Statistical Parametric Mapping) software package (Gaser et al. 2024) running under Matlab 2021a. The plasticity longitudinal preprocessing pipeline in CAT12 was used to derive morphometric features and appropriately account for the properties of the longitudinal MRI data. The processing included segmentation, bias correction, and spatial normalization. The individual regional GM volume was extracted from the Neuromorphometrics' probabilistic brain atlas (Neuromorphometrics Inc., https://www.neuromorphometrics.com/) as a default atlas in SPM/CAT12 environment, covering the entire brain cortex. Regional relative GM volume values (corrected for the total intracranial volume) were used as an outcome in the model described in the following sections.

### 2.4 Materials and Time‐Varying Predictors of Brain Changes

A wide range of predictors that could possibly affect the brain (e.g., physical environment, physiological, lifestyle, and psychological measures) were collected at each scanning time point and were included in the analysis. These include covariates about the participants' state during the scanning session, as well as their behavior and well‐being 24 h prior to the scan. The subjects' mood was measured with the Positive and Negative Affect Schedule (PANAS, Watson et al. 1988). Environmental variables, such as scanner room temperature, humidity and weather conditions, were also monitored. Furthermore, activity data (i.e., calorie expenditure, step count, and hours of sleep) were gathered using a Fitbit One activity tracker (Fitbit, San Francisco, USA), with data collected daily over a 24‐h period before MRI acquisition. Steroid hormone concentration was determined from saliva samples with Saliva ELISA kit (IBL‐International, using IBL Saliva Immunoassay −17ß‐Estradiol) and the IBL Saliva Testosterone Luminescence Immunoassay. A detailed description of all available variables can be found in Filevich et al. (2017). All the included predictor variables are available in the Table S1.

### 2.5 Statistical Analysis

The data analysis was conducted using R (Version 4.4.3., R Core Team 2021) and RStudio (Version: 2024.04.2 + 764). The quality of the preprocessed MRI data was examined through CAT12's structural image quality rating framework (SIQR; Dahnke et al. 2025). The images were of overall very good quality (mean SIQR = 1.70 ± 0.08, where SIQR rating of 0.5 represents the highest quality rating and 10.5 the lowest). Three scans were identified as outliers in terms of quality (visible motion artifacts with an SIQR of 1.91, 1.96, and 2.88; see example slices in Figure S3). However, in order to remain consistent with the approach of Karch et al. (2019), these scans were not excluded from the main analysis. Interested readers can refer to the Supporting Information, which presents the results of the analysis after excluding the quality outliers.

For the time‐varying covariates, we adopted the data cleaning procedure from Karch et al. (2019).

FitBit activity measurements from days leading up to the scanning session were averaged and matched with the corresponding questionnaire responses and the neuroimaging measurements from the day of the scan. A critical consideration was that FitBit activity data from the scanning day itself could not be directly associated with brain morphometric values on that day. Since participants were typically scanned in the morning, the activity data for the same day predominantly reflected postscan periods and, therefore, could not have influenced the imaging results. To address this, the activity data from scanning day *x* were included in the computation of the activity for the scanning day *x + 1*.

#### 2.5.1 Main Analysis Using sPLS

Identifying measurement co‐variation with time‐varying predictors could have been achieved in a mass‐univariate approach. However, given the considerable number of brain regions and predictors, we opted for a more sensitive multivariate approach that decomposes the entire correlation structure of the regional brain volumes and covariate feature sets into a few latent variables (further denoted components) of low rank.

More specifically, we employed multilevel sparse Partial Least Squares regression (sPLS) in the *MixOmics* package version 6.31.4 (Rohart et al. 2017) to examine which predictors were associated across sessions with relative GM volume in specific brain regions. sPLS is a multivariate statistical method and a variation of the Partial Least Squares (PLS) regression (Lê Cao and Welham 2021), which has previously been used in neuroimaging studies (e.g., Ziegler et al. 2013). PLS can be employed to investigate the relationship between two datasets by identifying the latent factors underlying their association (Wold et al. 1984). The method enables the analysis of noisy and collinear data, while simultaneously modeling multiple outcome variables (Lê Cao and Welham 2021; Wold et al. 1984). sPLS, as its extension, facilitates the same type of analysis. Moreover, as a sparse method, it also allows for variable selection, meaning it can identify the most relevant variables in the dataset while ignoring less important ones. This is done by applying LASSO (least absolute shrinkage and selection operator) penalisation on the loading vectors to select a smaller subset of variables that contribute most to explaining the relationships between predictor and outcome variables (Lee et al. 2011). Finally, the subset of variables is summarized into components (via projection) that in turn reflect the correlation structure of the features in a manner analogous to a principal component analysis but for multiple sources (such as regions and covariates) while optimizing sparse loadings for maximal co‐variance of projection scores. Each extracted component is therefore reflected by a latent score (from projection) for each of the two sources (X and Y) and a set of loadings (or weights) for each of the features analyzed.

Notable, in contrast to most previous PLS applications, in this study a multilevel analysis was applied to account for the longitudinal/repeated‐measures design. The multilevel approach separates two sources of variance (between‐ and within‐subject variance) and performs matrix deflation on the within‐person variance matrix (Lê Cao and Welham 2021; Liquet et al. 2012).

Furthermore, we set the mode of analysis to canonical (i.e., similar to canonical correlation analysis) to model symmetrical relationship between X (questionnaire and activity data: 72 variables) and Y (regional GMV: 122 variables). The optimal number of components and the loading variables per component were determined based on the initial tuning of the model in a 10‐fold cross‐validation approach based on the maximization of the correlation criterion.

#### 2.5.2 Complementary Analyses

To further elucidate the findings, we performed a series of complementary analyses.

Firstly, we conducted an sPLS analysis as described above on data acquired in 1 month (July 2013) to limit the effects of aging. One subject (Subject 4) was excluded from the analysis due to a reduced number of available scans that month. In total, 61 scans were used in this analysis (subject 1: *n* = 10; subject 2: *n* = 7; subject 3: *n* = 8; subject 5: *n* = 10; subject 6: *n* = 10; subject 7: *n* = 6; subject 8: *n* = 10).

Secondly, given the large importance of the data quality, we employed a multilevel canonical sPLS model, using questionnaire and activity data as predictors (*n* = 71 variables) and SIQR quality rating as the outcome. The analysis was performed on the dataset without the quality outliers. To help ease the interpretation of the results, we fixed the number of possible extracted predictor variables to 10.

## 3 Results

**FIGURE 1:**
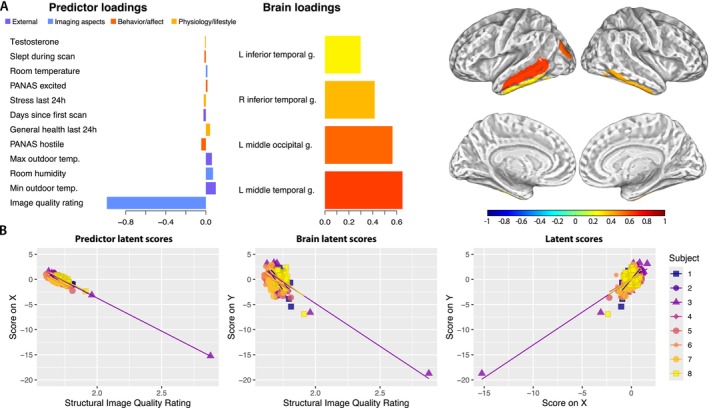
(A) The predictor and outcome loadings on the component extracted from the sparse PLS multilevel model (left) and the surface mapping of the loadings of brain regions from the Neuromorphometrics atlas associated with respective components (right). All presented weights were identified as nonzero by model optimization and are shown here in an ascending order of their absolute value. Structural image quality rating had the strongest impact on the extracted component, even in the case of a dataset with good image quality. (B) Predictor‐ and outcome‐related sPLS latent scores (X and Y) shown over the image quality rating (left and middle) and the relationship between both sPLS latent scores (right). We illustrate multilevel sPLS scores and relationships on an individual participant level to support transparency. Note the three outliers in image quality, which were removed for the supplementary analysis (Figure S1).

**FIGURE 2:**
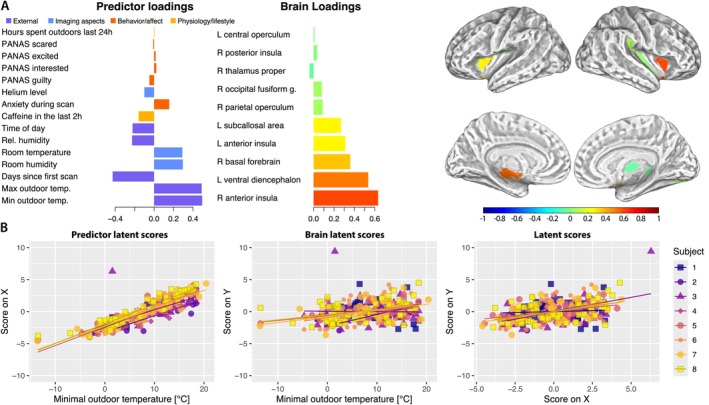
(A) Season‐related and temporal variables are most strongly associated with the extracted component, which mainly correlates with insular brain regions. (B) Predictor‐ and outcome‐related sPLS latent scores shown over the minimal outdoor temperature (left and middle) and the relationship between both sPLS latent scores for component 2 (right).

In order to identify the potential contributors to volumetric fluctuations over repeated measures, we focused on a set of variables including environmental/physical properties (indoor and outdoor humidity and temperature), acquisition and design parameters (image quality, time since baseline, time of start of scanning), physiological and lifestyle measures (hormone levels, caffeine intake, amount of physical activity), as well as psychological measures (affect score). We illustrate the extracted predictors and the brain variables from the main analysis (Figures 1 and 2), and provide the results of the complementary analysis (see Methods) in the following paragraphs. In what follows, we primarily focus the presentation on the variables most strongly associated with their respective components (i.e., with highest loadings) to improve the clarity of the results.

### 3.1 Main Analysis

The results of our main analysis showed that the extracted component 1 was largely associated with structural image quality ratings (Figure 1A left). This component was also found to associate with outdoor temperatures and room humidity (Figure 1B left and middle). Brain regions where GM volume was associated with the extracted component included the *left middle temporal gyrus, left middle occipital gyrus*, as well as the *left and right inferior temporal gyri* (Figure 1A right). Furthermore, since sPLS finds latent components that maximize the covariance between X and Y, each component explains a different proportion of variance in both datasets. The first component explained 2.38% of the variance in the predictor dataset and 31.98% in the GM volume data variability.

Component 2 accounted for 7.25% of the variance within the predictors and 6.34% of the total variance of the GM volume and is associated with outdoor temperatures (Figure 2B left and middle) and the time passed since the baseline scan. Additionally, MR room humidity and temperature, as well as the start time of the scanning session, were included in this component, which correlates with the gray matter volume in the *right* and *left anterior insula, left ventral diencephalon*, and the *right basal forebrain*. Changes in GM volume in the right anterior insula over time are shown in the Figure S2. The associations within the data are summarized in the Figure S4.

### 3.2 Complementary Analysis I

**FIGURE 3:**
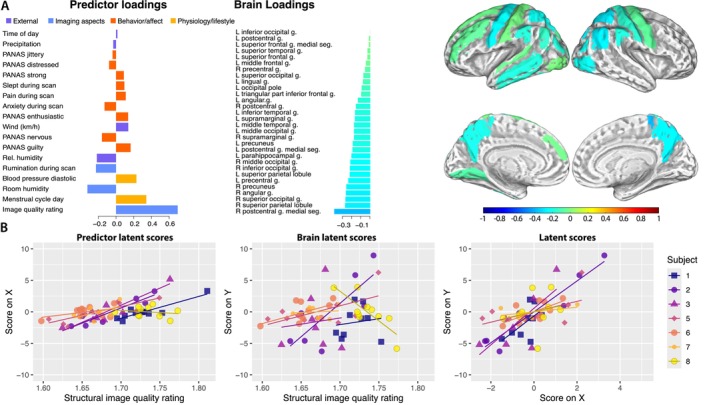
(A) The results of the complementary analysis showing the brain changes within 1 month. Image quality, the day of the menstrual cycle, and MR room humidity are the most important predictors associated with the extracted component, which moderately correlates with multiple regions across the left hemisphere and the right parietal and occipital regions. It is important to note that a higher image quality rating indicates lower image quality. (B) sPLS‐derived latent scores show a positive association with the image quality rating.

The complementary analysis was performed on 61 scans acquired in one summer month with the greatest amount of scans per person. The following predictors of brain gray matter volume changes were extracted in this analysis: image quality, the day of the menstrual cycle, MR room humidity, diastolic blood pressure, rumination during scan, relative outdoor humidity, and various behavioral and affective variables during scanning session (e.g., anxiety, physical pain, and sleeping in the scanner). The pattern of GM volume changes within 1 month is dispersed across the left hemisphere and the right parietal and occipital parts of the brain, most evidently in the *medial segment of the right postcentral gyrus, the right superior parietal lobule, the right superior occipital gyrus, the right angular gyrus, the left precuneus* etc. The extracted component explained 5.3% of the variance in the predictor dataset and 30.4% of the variability in regional GM volume (Figure 3).

### 3.3 Complementary Analysis II

**FIGURE 4:**
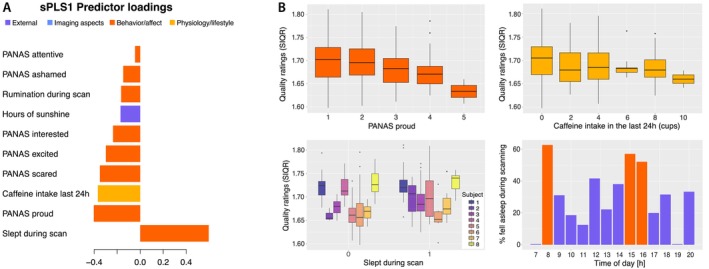
(A) The results of the complementary sPLS1 analysis selecting the 10 most influential variables associated with the structural image quality rating, where a higher rating indicates a lower image quality. The most influential variable associated with lower quality of the images was the information on whether the person slept during the scanning session. Other influential factors are positive and negative affective variables, caffeine intake in the 24 h prior to the scan, hours of sunshine, and rumination during scanning session. Please note that scans containing motion artifacts were excluded from the analysis, and that the image quality of this dataset is high overall. (B) The relationship between image quality ratings and their three most important predictors. Again, please note that the quality of these scans was good and that the range of possible image quality ratings spans from 0.5 (best) to 10.5 (worst). On average, image quality was higher when participants reported feeling proud or had drunk more cups of coffee during the day (top). Within‐subject variability in quality ratings was greater when participants fell asleep during scanning (bottom left). Furthermore, it appears that participants reported falling asleep during scanning differently at various times of day (bottom right).

Given the importance of the MR image quality in the first component, we conducted an sPLS1 analysis investigating the association of various predictor variables and the structural image quality rating. The most influential predictors were information on whether a person fell asleep during the scanning session, PANAS affective variables (e.g., proud, scared, excited, interested, attentive), caffeine intake in the previous 24 h, hours of sunshine, and rumination during scan (Figure 4).

## 4 Discussion

Investigating the factors that contribute to intraindividual variability in brain structure over a short period of time is essential for understanding how our brain works, but also for improving the reliability of MRI measurements. In the present study, we used multilevel sparse partial least squares regression to analyse a densely sampled MRI dataset, with a focus on trends and fluctuations in relation to a wide range of physiological, psychological, lifestyle, environmental, and acquisition‐related parameters over 25 weeks. Our results showed that the changes in regional GM volume estimations are primarily driven by variation in structural image quality, but also other temporal‐ and season‐related factors. To avoid misinterpreting potential noise in the results, the following discussion will focus on the most prominent extracted factors and regions per analysis.

### 4.1 Main Analysis

Previous study on within‐person variance in brain morphometry in the Day2day dataset by Karch et al. (2019) has identified associations between *Days since the first scan* and *Time of da*y with FreeSurfer‐derived estimates of total brain GM volume. Additionally, they found (weaker) evidence for associations with *Number of steps* and *Testosterone levels*. The present study extends their investigation by employing a multilevel sparse partial least squares analysis on regional GM volume. Despite different preprocessing software and analytical methods, our study corroborated some of their findings while providing further insights into the contribution of image quality and the factors affecting it. Similarly to Karch et al. (2019), we could observe the prominence of *Temperature* and *Days since the first scan* in predicting within‐person differences in brain measures, especially in the second component orthogonal to the image quality‐related component.

Although Karch et al. (2019) included an aspect of image quality in their analyses (i.e., FreeSurfer's number of defect holes), this predictor did not appear as important in their results. While reflecting the quality of the segmentation, the number of defect holes represents only one aspect of image artifacts. Further metrics, derived from image quality‐control tools, such as MRIQC (Esteban et al. 2017), CAT12 (Dahnke et al. 2025) or others, can provide a more comprehensive evaluation of image quality. Interestingly, even in a high‐quality MRI dataset like the Day2day, image quality was the most prominent factor contributing to short‐term changes in gray matter volume also when the scans with motion artifacts were excluded (see the supplementary analysis).

The first extracted component, predominantly representing the structural image quality, showed an association with the left‐lateralized middle temporal and middle occipital gyri, as well as the left and right inferior temporal gyri. Although there is no clear explanation for our results, it has been shown that temporal brain areas are subject to micromovement artifacts (Alexander‐Bloch et al. 2016), while the occipital region may reflect barely visible fluctuations associated with bias correction or skull stripping (Acosta‐Cabronero et al. 2008). The partial lateralization observed in our results may be associated with the left hemisphere's greater vascularization (Jansen Van Vuuren et al. 2024), which could also affect the processing and variability in that region.

The second extracted component indicates that other prominent variables driving the short‐term changes in the gray matter volume were the outdoor temperatures and days since baseline. Since the Day2day data collection started in summer and concluded in winter, these results might reflect the passage of time and the aging process of the participants, even though they were comparably young (age range: 24–32 years). The observed negative correlation between *Days since first scan* and GM volume, along with the positive correlation between *Minimum* and *Maximum Temperature* and GM volume, could suggest the interpretation of GM volume decline related to increasing age.

However, the spatial pattern of gray matter loss was atypical of age‐related processes, visible predominantly in the insular cortex, ventral diencephalon, basal forebrain, and the subcallosal area. The observed pattern could be indicative of other processes, such as habituation or seasonal effects. The anterior insula and ventral diencephalon form a part of the salience network, responsible for processing and responding to homeostatically relevant signals (Seeley et al. 2007; Seeley 2019; Uddin et al. 2017). Together with the basal forebrain, a key contributor to sustained attention (Sarter et al. 2001), these regions show a decrease of gray matter volume with the progression of the study. Our results could therefore indicate participants' flexible adaptation to the scanning. On the other hand, an increasing body of research reports seasonal effects on the brain (Xu et al. 2023; Zhang et al. 2023). However, these studies predominantly observe the effects in the somatomotor networks and the posterior insula. Moreover, other studies have reported inconsistent evidence for the association of temperature with brain volume (Majrashi and Alyami 2022). Due to the limited study time of Day2day, season‐ and age‐related features cannot be separated, and further research is needed to better understand these effects.

### 4.2 Complementary Analysis II: Monthly Plasticity

We conducted our first complementary analysis to better understand the factors and changes within a shorter time span, where the potential effect of aging would be less apparent. Not surprisingly, image quality was again the leading factor associated with estimated brain changes. Nevertheless, additional factors associated with physiology were identified in this analysis. Although we did not replicate the findings from Karch et al. (2019), who found evidence of the effects of testosterone on the within‐person variance, our analysis revealed a positive association between the day of the menstrual cycle and the extracted component. The influence of the menstrual cycle on the brain has received increasing attention in recent years (Pritschet et al. 2020; Heller et al. 2025). Lisofsky et al. (2015) have reported changes in the volume of the hippocampus from early to late follicular phase, and Heller et al. (2025) have shown a dispersed effect of gonadal hormones on the brain during the menstrual cycle. Although the pattern found in our study is present across a large part of the brain, we cannot draw any definitive conclusions about how the day of the menstrual cycle affects its structure.

Nevertheless, contrary to the existing evidence on the influence of hydration and caffeine intake on the brain (e.g., Duning et al. 2005; Field et al. 2003; Kempton et al. 2009; Koppelstaetter et al. 2010), our short‐term analysis did not identify such effects. The results of this study are to some extent in line with the findings of a precision imaging study by Zahid et al. (2022), who have found no significant differences in regional brain volume due to physiological factors, such as caffeine intake, blood pressure, hydration, or time of day. However, we did observe an association between changes in diastolic blood pressure and changes in the brain. Given that our sample of young adults is not hypertensive and mainly consists of women, these results may be associated with variations of blood pressure during the menstrual cycle (Greenberg et al. 1985). Finally, it is important to note that the actual signal in this analysis cannot be separated from the image quality.

### 4.3 Complementary Analysis II: Factors Affecting Image Quality

In our final complementary analysis, we focused on identifying the factors that most contribute to differences in image quality. The most influential variable associated with worse image quality was the information on whether the person slept during the scanning session. Muscle relaxation while falling asleep can cause motion artifacts that affect subsequent processing and confound the results of the analyses (Alexander‐Bloch et al. 2016; Nárai et al. 2022; Stucht et al. 2015), typically reducing the estimated gray matter volume and thickness (Reuter et al. 2015; Dahnke et al. 2025). Other influential factors included positive and negative emotional variables, such as feeling proud, scared, excited, ashamed, or attentive, as well as reporting rumination during scanning. These affective states can help people remain awake during scanning and potentially reduce motion artifacts. However, elevated emotional arousal independent of affective valence can potentially also induce movement.

Perhaps more surprisingly, although we did not find a strong effect of caffeine consumption in the brain‐related analyses, caffeine intake 24 h prior to the scanning session was identified among important predictors of image quality. This result is probably due to reduced sleepiness during the scan, which is caused by caffeine's stimulating effect (Barry et al. 2005; Fiani et al. 2021). Similarly, the increased length of daylight hours in summer is associated with reduced subjective sleepiness (Danilenko et al. 2021) and was also identified as a factor affecting image quality in our study. However, Zhang et al. (2023) have found an association between greater head motion in fMRI and the daylength, indicating a need for further research on this topic.

The results of the Karch et al. (2019) study on the Day2Day dataset, and to some extent our own, have also highlighted the importance of the time of day at which scanning is started. Several studies have found a significant diurnal effect on brain volume changes, with larger volumes in the morning (Nakamura et al. 2015; Murata et al. 2024). While Nakamura et al. (2015) speculatively explain their findings with hydration status, distribution of fluid within the body, or heating of the scanner coil throughout the day, Mure et al. (2018) consider daily rhythms of gene expression as the underlying mechanisms.

Based on the results obtained in the present study, it would be advisable to consider time of image acquisition to account for day‐to‐day brain volume fluctuation in future neuroimaging studies, not only to account for potential physiological fluctuations, but also to help the participants stay awake and reduce movement during the scanning session. It has been shown that engagement during image acquisition decreases the amount of motion (Huijbers et al. 2017) and our results suggest that mental or emotional activity during scanning is associated with better image quality.

### 4.4 Strengths and Limitations

Given the exploratory nature of this study, its limited sample size and gender imbalance, the findings should be interpreted with caution. Further research is needed to explore the generalizability of our results in more diverse datasets. It is important to note that the extracted components explain only a small amount of the total variability in the dataset and that there is a possibility of model over‐fitting. While the extracted components contribute most to explaining the relationships between the predictor and the brain variables, the data and results are highly heterogeneous. Further research is needed to investigate more specific brain–behavior associations. Future studies could benefit from including breathing motion and cardiac pulsation parameters, which are used for prospective motion correction in high resolution imaging (Herbst et al. 2014; Stucht et al. 2015). Additionally, many of the covariates included in the present study were based on self‐report, which may have affected our findings. Prospective studies could therefore benefit from measuring relevant variables more objectively (e.g., hydration status). Furthermore, adopting a longitudinal design spanning more than 6 months could help to distinguish the effects of time and seasonality.

Nevertheless, the present study expanded upon the analysis of Karch et al. (2019), showing that variations in structural image quality can account for a large proportion of short‐term gray matter volume fluctuations, even within a high‐quality dataset. Additionally, our results suggest that, if measures to ensure high image quality have been taken, factors associated with physiology (e.g., hydration status, caffeine intake) that are not of interest to a particular study can be partially disregarded. Future (longitudinal) MRI studies could benefit from considering the start of image acquisition to ensure that participants are well rested and alert, as this can influence image quality.

## 5 Conclusion

This study aimed to partially replicate and expand upon the findings of the previous analysis of the Day2day dataset by Karch et al. (2019). We used a different preprocessing pipeline and applied a multi‐level sparse PLS model to identify factors affecting short‐term intra‐individual changes in regional GM volume of the human brain. Similarly to Karch et al. (2019), we could observe the effect of time on the brain of young adults over 6 months. Moreover, our analyses have shown that image quality aspects beyond (visible) motion artifacts largely affect the results, and that factors that influence image quality are mainly associated with participants' attentiveness during scanning.

## Author Contributions

**Polona Kalc:** conceptualization, formal analysis, writing – original draft, writing – review and editing, visualization. **Mayla ter Veer:** formal analysis, writing – original draft, visualization. **Robert Dahnke:** software, conceptualization, visualization, writing – review and editing. **Gabriel Ziegler:** writing – review and editing. **Simone Kühn:** resources, writing – review and editing. **Christian Gaser:** supervision, funding acquisition, writing – review and editing.

## Funding

The study was supported by Carl Zeiss Stiftung as a part of the IMPULS project (IMPULS P2019‐01‐006), the Federal Ministry of Science and Education (BMBF) under the frame of ERA PerMed (Pattern‐Cog ERAPERMED2021‐127), the Marie Skłodowska‐Curie Innovative Training Network (SmartAge 859890 H2020‐MSCA‐ITN2019) and the European Union (ERC‐2022‐CoG‐BrainScape‐101086188). Views and opinions expressed are however those of the authors only and do not necessarily reflect those of the European Union or the European Research Council Executive Agency (ERCEA). Neither the European Union nor the granting authority can be held responsible for them.

## Conflicts of Interest

The authors declare no conflicts of interest.

## Supporting information


**Table S1:** A list of included predictor variables with descriptions and the amount of missing data. The comments, labels, and assessment period are adapted from Filevich et al. (2017) and Karch et al. (2019).
**Figure S1:** (A) The predictor and outcome loadings on the only component extracted from the sparse PLS multilevel model applied to the dataset without scans with motion artifacts. Even in the case of a dataset with good image quality and no outliers with motion artifacts, structural image quality rating had the strongest impact on the extracted component. The latter explained 4.4% of the variance in the predictor dataset and 25.8% in the GM volume data variability. While extracting the second component, the warning about the algorithm not converging appeared. (B) Predictor‐ and outcome‐related sPLS latent scores (X and Y) shown over the image quality rating (left and middle) and the relationship between both sPLS latent scores (right).
**Figure S2:** Changes in relative GM volume of the right anterior insula over time.
**Figure S3:** A distribution of image quality metrics with three identified outliers and the corresponding MRI images. Image C shows moderate motion artifacts, while images A and B show light motion artifacts.

**Figure S4:** A dendrogram plot of associations within the dataset.

## Data Availability

The Day2day data is available freely on request from Prof. Dr. Simone Kühn. The code to replicate the findings is available at Figshare (DOI: 10.6084/m9.figshare.28839635).
