# Supplementary material for: Factors Contributing to Short‐Term Structural Variability in a Longitudinal MRI Dataset
Source: Hum Brain Mapp. 2026 Mar 12;47(4):e70500. doi: 10.1002/hbm.70500 (PMC13093422; doi:10.1002/hbm.70500)
Supplement: Supplementary file 1 — Table S1: A list of included predictor variables with descriptions and the amount of missing data. The comments, labels, and assessment period are adapted from Filevich et al. (2017) and Karch et al. (2019). Figure S1: (A) The predictor and outcome loadings on the only component extracted from the sparse PLS multilevel model applied to the dataset without scans with motion artifacts. Even in the case of a dataset with good image quality and no outliers with motion artifacts, structural image quality rating had the strongest impact on the extracted component. The latter explained 4.4% of the variance in the predictor dataset and 25.8% in the GM volume data variability. While extracting the second component, the warning about the algorithm not converging appeared. (B) Predictor‐ and outcome‐related sPLS latent scores (X and Y) shown over the image quality rating (left and middle) and the relationship between both sPLS latent scores (right). Figure S2: Changes in relative GM volume of the right anterior insula over time. Figure S3: A distribution of image quality metrics with three identified outliers and the corresponding MRI images. Image C shows moderate motion artifacts, while images A and B show light motion artifacts. [file HBM-47-e70500-s001.docx]

**Supplementary material**

**Table S1**

A list of included predictor variables with descriptions and the amount of missing data. The comments, labels, and assessment period are adapted from Filevich et al. (2017) and Karch et al. (2019).

| **Variable** | **Label** | **Description &**  **descriptive statistics** | **Assessment Period** | **Missing Data** |
| --- | --- | --- | --- | --- |
| **External** | | | | |
| Days since the first scan of this person | Days Since First Scan | M=89.02, SD=61.60, min=0, max=232 | Scan Session | 0 |
| Time of start of the scanning session (h) | Time of Day | M=11.75, SD=3.45, min=7, max=20 | Scan Session | 13 (4.32%) |
| Minimum outdoor temperature on the day of the scan (°C) | Min outdoor temp. | M=8.31, SD=6.38, min=-13.6, max=20.4 | Scan Session | 0 |
| Maximum outdoor temperature on the day of the scan (°C) | Max outdoor temp. | M=16.58, SD=8.36, min=-8.4, max=33.2 | Scan Session | 0 |
| Wind (km/h) | Wind (km/h) | M=13.22, SD=4.00, min=6, max=25 | Scan Session | 0 |
| Precipitation (mm) | Precipitation (mm) | M=1.91, SD=5.01, min=0, max=33.3 | Scan Session | 0 |
| Hours of sunshine on the day of the scan | Hours of Sunshine | Mdn=3.9, IQR=8.75, min=0, max=15.8 | Scan Session | 0 |
| Atmospheric pressure (hp) | Atm. pressure | M=1006.50, SD=7.92, min=982.48, max=1023.82 | Scan Session | 0 |
| Relative humidity (%) (daily average) | Rel. humidity | M=77.90, SD=12.36, min=49.96, max=99 | Scan Session | 0 |
| **Scanner/imaging characteristics** | | | | |
| MR room temperature (°C) | Room Temperature | M=21.46, SD=0.98, min=19.2, max=24.2 | Scan Session | 2 (0.66%) |
| MR room Humidity (%) | Room Humidity | M=51.51, SD=8.10, min=32.9, max=70.9 | Scan Session | 4 (1.03%) |
| MR helium level (%) | Helium Level | M=82.20, SD=11.82, min=61.8, max=99.2 | Scan Session | 1 (0.33%) |
| Structural Image Quality Rating (SIQR) | Image quality rating | Continuous measure estimated in CAT12: 0.5 - best quality to 10.5 - worst quality  M=1.69, SD=0.04, min=1.60, max=1.81 | Scan Session | 0 |
| **Behavioural and affective variables during scanning** | | | | |
| Slept during scanning | Slept during scan | binary (1 - yes, 0 - no)  1: 38% | Scan Session | 7 (2.32%) |
| Rumination during scanning | Rumination during scan | 1–6 Likert scale  M=1.83, SD=1.50, min=0, max=6 | Scan Session | 3 (0.99%) |
| Anxiety during scanning | Anxiety during scan | 1–6 Likert scale  M=0.51, SD=0.91, min=0, max=5 | Scan Session | 1 (0.33%) |
| Physical pain during scanning | Physical pain | 1–6 Likert scale  Mdn=1, IQR=2, min=0, max=5 | Scan Session | 0 |
| Positive and Negative Affect Schedule (PANAS) | PANAS_[item] | Assessed during the scanning.  The PANAS questionnaire measures 2 orthogonal components of affect | Scan Session | 0 |
| **Physiological and lifestyle variables** | | | | |
| Day of menstrual cycle | Menstrual cycle day | Mdn=13, IQR=13.75, min=1, max=35 | Scan Session | 51 (16.9%) |
| Caffeine intake in the last 24 h | Caffeine Intake Last 24 h | In an equivalent number of cups of coffee  Mdn=2, IQR=3, min=0, max=10 | 24 h | 0 |
| Caffeine intake in the last 2 h | Caffeine Intake Last 2 h | In an equivalent number of cups of coffee  Mdn=0, IQR=1, min=0, max=3 | 2 h | 6 (1.99%) |
| Cocoa intake (%) in the last 24 h | Cocoa Intake Last 24 h | Mdn=0, IQR=0, min=0, max=200 | 24 h | 25 (8.31%) |
| Cocoa intake (%) in the last 2 h | Cocoa Intake Last 2 h | Mdn=0, IQR=30, min=0, max=70 | 2 h | 18 (5.98%) |
| Sweets intake in the last 24 h | Sweets intake Last 24h | Mdn=2, IQR=3, min=0, max=6 | 24h | 12 (3.99%) |
| Chocolate intake (g) in the last 24 h | Chocolate intake Last 24h | Mdn=0, IQR=30, min=0, max=150 | 24h | 61 (20.27%) |
| Chocolate intake (g) in the last 2 h | Chocolate intake Last 2h | Mdn=0, IQR=0, min=0, max=50 | 2 h | 60 (19.93%) |
| Weight (kg) | Weight | Weighed fully dressed, without shoes.  M=64.85, SD=5.67, min=57.8, max=83 | Scan Session | 2 (0.66%) |
| Alcohol intake in the last 24 h | Alcohol Intake Last 24 h | Number of alcoholic drinks  Mdn=0, IQR=0, min=0, max=8 | 24 h | 0 |
| Cigarettes smoked in the last 24 h | Cigarettes Last 24 h | Mdn=0, IQR=0, min=0, max=1 |  | 0 |
| Liquid intake in the last 24 h (l) | Liquid Intake Last 24 h | M=2.19, SD=0.94, min=0, max=5 | 24 h | 0 |
| Blood pressure (mmHg, systolic and diastolic) | Blood Pressure Systolic  Blood Pressure Diastolic | M=115.06, SD=10.95, min=86, max=162  M=73.71, SD=8.09, min=56, max=112 | Scan Session | 14 (4.65%), 12 (3.99%) |
| Estradiol in (pg/mL) | Estradiol | Measured using saliva samples  Mdn=41, IQR=44.25, min=2, max=204 | Scan Session | 81  (26.91%) |
| Testosterone (pg/mL) | Testosterone | Measured using saliva samples  Mdn=140, IQR=248.75, min=14, max=1094 | Scan Session | 77 (25.58%) |
| General health rating | General health | Subjective rating of the last 24 h, 1–6 Likert scale  Mdn=5, IQR=1, min=0, max=6 | Scan Session | 0 |
| General stress subjective rating | Stress Last 24h | Subjective rating of the last 24 h on 1–6 Likert scale  M=2.71, SD=1.41, min=0, max=6 | 24 h | 0 |
| Ease of concentration | Ease of concentration | Subjective rating of the last 24 h on 1–6 Likert scale  M=3.63, SD=1.33, min=0, max=6 | 24 h | 0 |
| Hours of work in the last 24h | Hours work Last 24h | Self-report  M=7.35, SD=3.38, min=0, max=15 | 24h | 0 |
| Hours of free time in the last 24 h | Hours free Last 24h | Self-report  Mdn=4, IQR=3, min=0, max=24 | 24h | 0 |
| Hours of sport in the last 24 h | Hours sport Last 24h | Self-report  Mdn=0, IQR=0.6, min=0, max=5 | 24h | 0 |
| Hours spent directly interacting with electronic devices in the last 24 h | Hours screen Last 24h | Self-report  Mdn=6, IQR=7, min=0, max=15 | 24h | 0 |
| Hours spent outdoors in the last 24 h | Hours outdoors Last 24h | Self-report  Mdn=1, IQR=1.5, min=0, max=8 | 24h | 0 |
| Hours spent in active social interaction in the last 24 h | Hours active social interaction Last 24h | Self-report  M=5.03, SD=2.84, min=0, max=15 | 24h | 0 |
| Hours spent in passive social interaction in the last 24 h | Hours passive social interaction Last 24 h | Self-report  Mdn=3, IQR=4, min=0, max=24 | 24h | 0 |
| Sleep quality last night | Sleep quality | 1–6 Likert scale, with its extremes specified as 1: “Very badly” to 6: “Very well”  Mdn=4, IQR=2, min=0, max=6 | 24h | 0 |
| Can remember dreams from previous night | Remember dreams | Binary variable (0/1), 1: 34% | 24h | 8 |
| Frequency of day dreams (mindwandering) in the last 24 h | Daydream Last 24h | 1–6 Likert scale  Mdn=1, IQR=1, min=0, max=6 | 24h | 7 (2.33%) |
| Number of stories climbed | Number of climbed stories | Measured with a FitBit© activity tracker.  Averaged over the days from the previous scanning session to the current.  Mdn=21, IQR=10.26, min=2, max=146.66 | Days between two sessions | 10 (3.32%) |
| Distance walked | Distance walked | Measured with a FitBit© activity tracker.  Averaged over the days from the previous scanning session to the current.  M=6.63, SD=2.31, min=0.52, max=16.33 | Days between two sessions | 10 (3.32%) |
| Number of steps taken | Steps | Measured with a FitBit© activity tracker. Averaged over the days from the previous scanning session to the current.  M=9122.81, SD=3182.87, min=739, max=20403.5 | Days between two sessions | 10 (3.32%) |
| Calories burned | Calories burned | Measured with a FitBit© activity tracker. Averaged over the days from the previous scanning session to the current.  M=2162.94, SD=290.42, min= 1423, max=3389 | Days between two sessions | 10 (3.32%) |
| Time went to bed the previous night | Bedtime | Measured with a FitBit activity tracker and completed manually in case of omission.  M=23:26:40, SD=01:33:45, min= 00:00:00, max=23:59:00 | 24h | 132 (43.85%) |
| Time spent in bed the previous night (min) | Time slept | Measured with a FitBit activity tracker and completed manually in case of omission.  M=439.867, SD=69.24, min=246.5, max=818 | 24h | 134 (44.52%) |

**Results from the dataset without quality outliers**

**~~
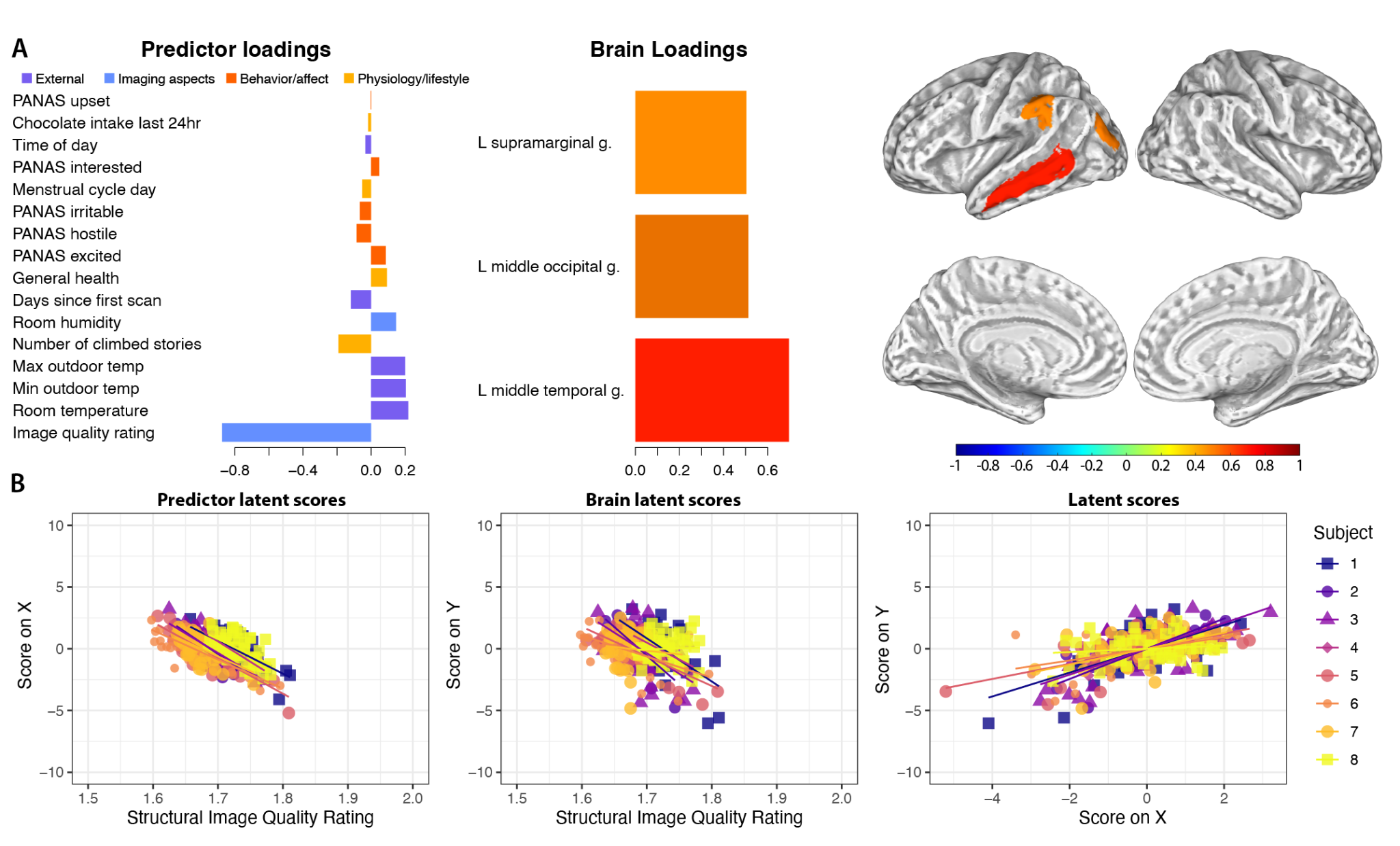
~~**

**Figure S1**

**A)** The predictor and outcome loadings on the only component extracted from the sparse PLS multilevel model applied to the dataset without scans with motion artifacts. Even in the case of a dataset with good image quality and no outliers with motion artifacts, structural image quality rating had the strongest impact on the extracted component. The latter explained 4.4% of the variance in the predictor dataset and 25.8% in the GM volume data variability. While extracting the second component, the warning about the algorithm not converging appeared. **B)** Predictor- and outcome-related sPLS latent scores (X and Y) shown over the image quality rating (left & middle) and the relationship between both sPLS latent scores (right).

**Changes in the volume of the right anterior insula**


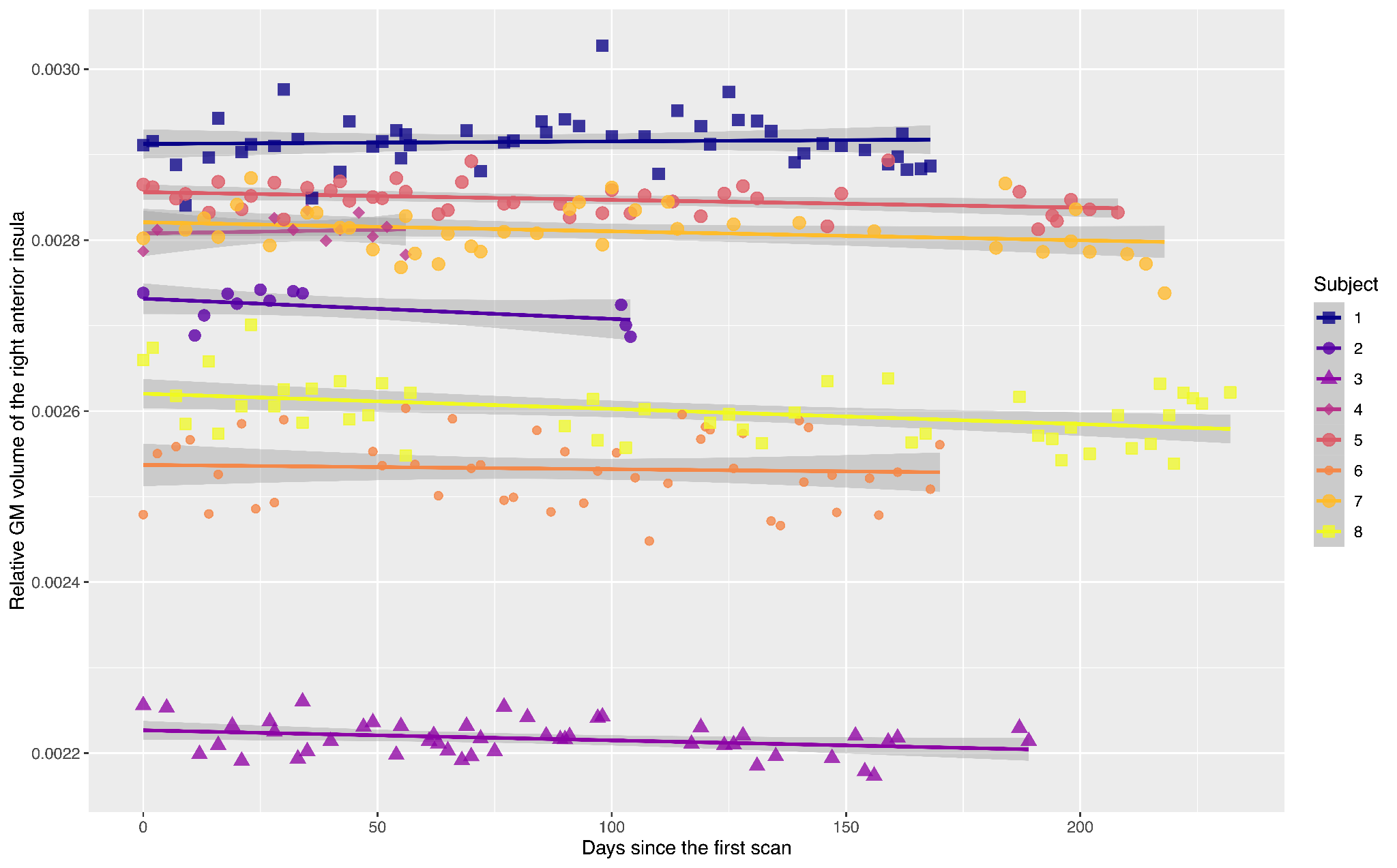


**Figure S2.** Changes in relative GM volume of the right anterior insula over time.

**Image quality ratings distribution**

**
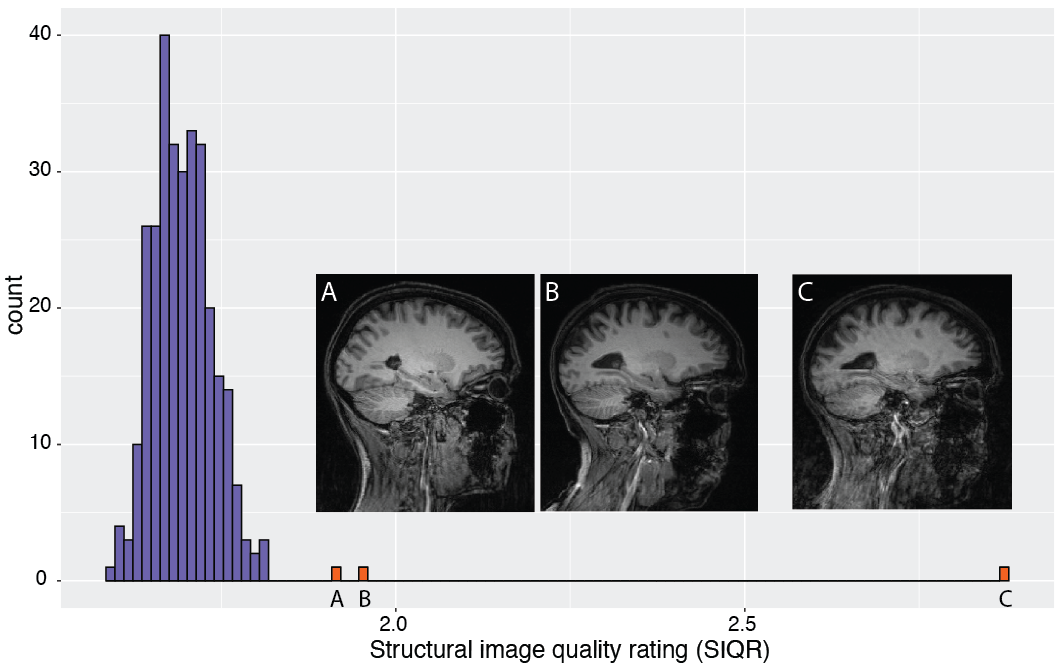
**

**Figure S3.** A distribution of image quality metrics with three identified outliers and the corresponding MRI images. Image C shows moderate motion artefacts, while images A and B show light motion artefacts.
