# Supplementary figures and images for: Factors Contributing to Short‐Term Structural Variability in a Longitudinal MRI Dataset
Source: Hum Brain Mapp. 2026 Mar 12;47(4):e70500. doi: 10.1002/hbm.70500 (PMC13093422; doi:10.1002/hbm.70500)

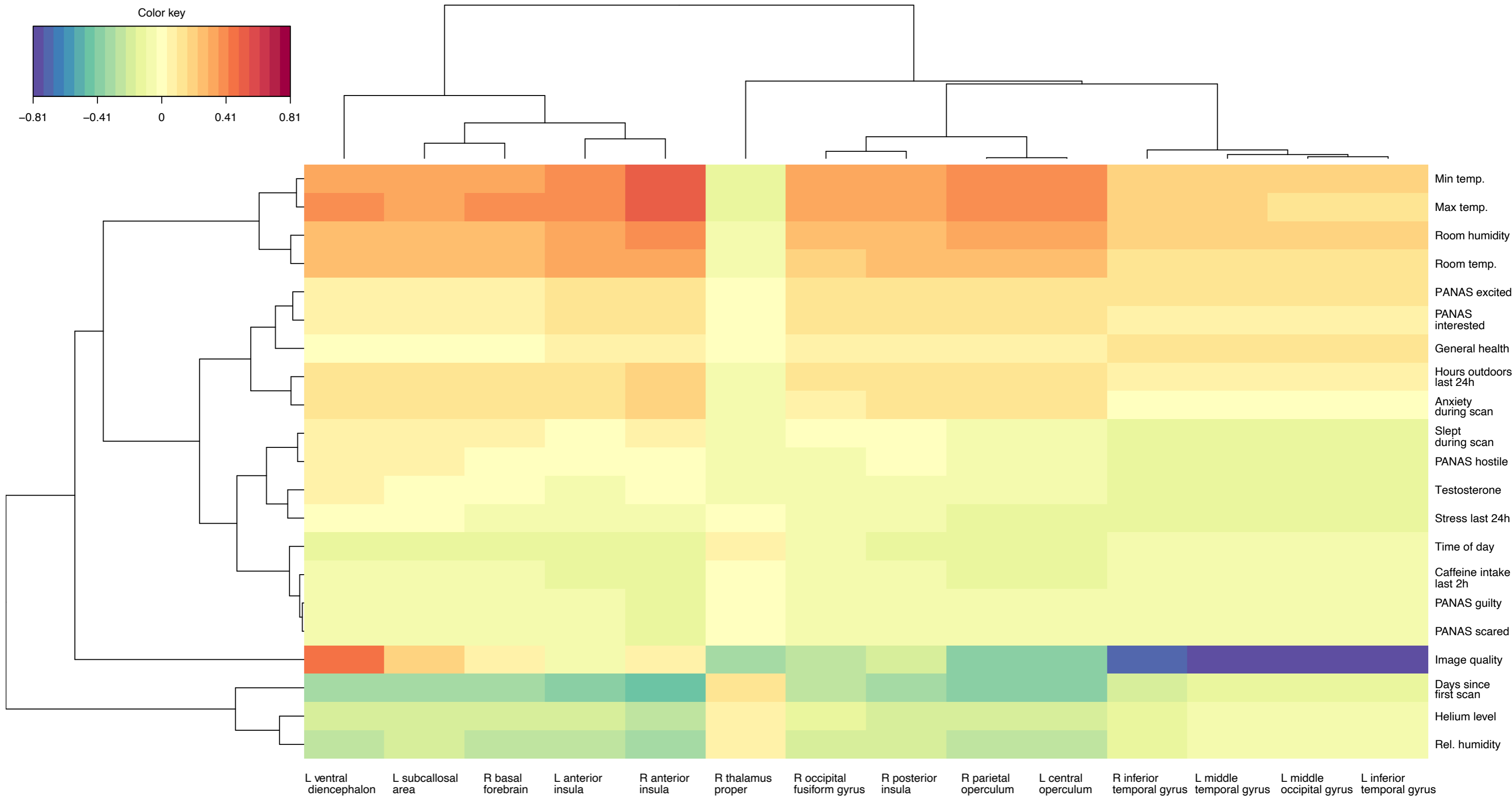

Supplement: Supplementary file 2 — Figure S4: A dendrogram plot of associations within the dataset. [file HBM-47-e70500-s002.pdf]
